# Human brains preserve in diverse environments for at least 12 000 years

**DOI:** 10.1098/rspb.2023.2606

**Published:** 2024-03-20

**Authors:** Alexandra L. Morton-Hayward, Ross P. Anderson, Erin E. Saupe, Greger Larson, Julie G. Cosmidis

**Affiliations:** ^1^ Department of Earth Sciences, University of Oxford, Oxford, UK; ^2^ Target Discovery Institute, University of Oxford, Oxford, UK; ^3^ All Souls College, University of Oxford, Oxford, UK; ^4^ Palaeogenomics and Bio-Archaeology Research Network, School of Archaeology, University of Oxford, Oxford, UK

**Keywords:** bioarchaeology, forensic anthropology, soft tissue preservation, taphonomy, diagenesis, brain

## Abstract

The brain is thought to be among the first human organs to decompose after death. The discovery of brains preserved in the archaeological record is therefore regarded as unusual. Although mechanisms such as dehydration, freezing, saponification, and tanning are known to allow for the preservation of the brain on short time scales in association with other soft tissues (≲4000 years), discoveries of older brains, especially in the absence of other soft tissues, are rare. Here, we collated an archive of more than 4400 human brains preserved in the archaeological record across approximately 12 000 years, more than 1300 of which constitute the only soft tissue preserved amongst otherwise skeletonized remains. We found that brains of this type persist on time scales exceeding those preserved by other means, which suggests an unknown mechanism may be responsible for preservation particular to the central nervous system. The untapped archive of preserved ancient brains represents an opportunity for bioarchaeological studies of human evolution, health and disease.

## 1. Background

Since the mid-17th century, more than 4400 human brains have been unearthed from the last 12 000 years of the archaeological record, over 1300 of which are preserved among otherwise skeletonized remains. Despite this volume of finds, the perception remains that preserved brains represent ‘unique’ or ‘extremely rare’ discoveries [1]. Human soft tissues are understood to persist through time by well-characterized mechanisms of preservation such as dehydration, freezing and tanning, brought about by anthropogenic (i.e. the result of deliberate human intervention) or naturally occurring factors. Thus, it is not surprising that the brain endures alongside other internal organs where there is extensive soft tissue preservation. Brains are found in the desiccated remains of desert burials [2], the frozen corpses of mountain passes [3], and the tanned bog bodies of low-lying wetlands [4]. However, the preservation of brains in the absence of other soft tissues—for example, among ancient human bones dredged from a swampy pond [5]—is unexpected, poorly reported, and represents an untapped source of bioarchaeological information.

Despite the prospective palaeobiological applications, no comprehensive or systematic effort has been made to collectively investigate preserved brains for factors that might explain why this organ persists when other soft tissues do not. At the dawn of the 20th century, anatomist and Egyptologist Elliot Smith (1871–1937) derided the ‘scanty literature’ on preserved brains, arguing that ‘almost every archaeologist who has excavated … is aware of the fact that the brain is preserved in the crania of a very large proportion’ of human remains disinterred [6]. To test the long-held hypothesis that human brain preservation is a rare phenomenon, we compiled an archive of preserved human brains in the archaeological record. We statistically analysed this body of data to establish their prevalence, persistence, and diversity of preservation types, described the nature of differentially preserved nervous tissues, and quantified their global and temporal distribution. We then discuss the value of leveraging preserved human brains for studies of palaeopathology and archaeogenetics.

## 2. Results

**Figure 1.**
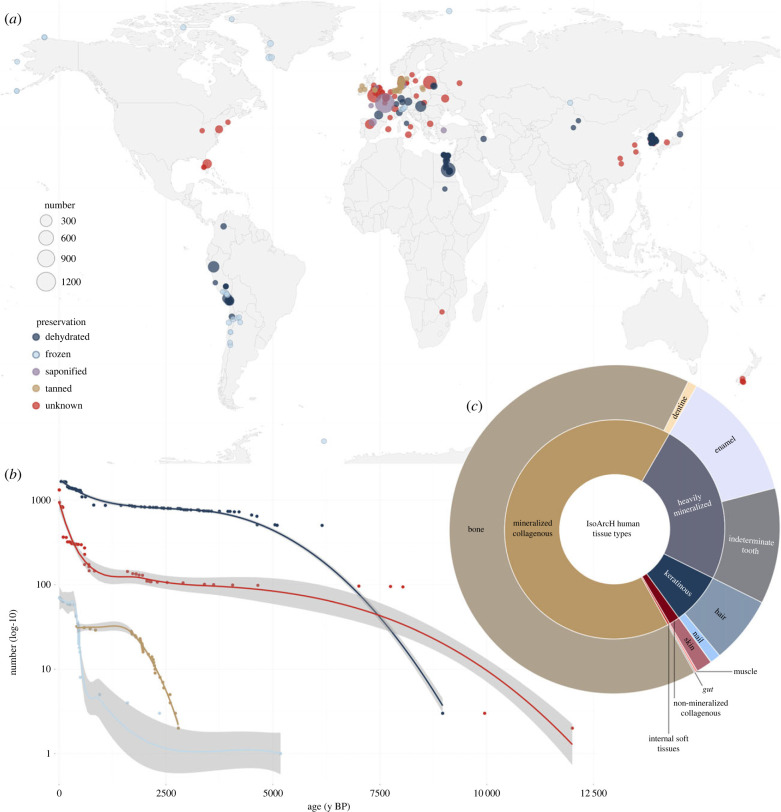
Distribution and frequency of preserved human brains by type, compared with soft tissues in the IsoArcH repository. (*a*) Global distribution of preserved human brains by type: dehydrated (*n =* 1667; 37.8%), frozen (*n =* 70; 1.6%), saponified (*n =* 1308; 29.7%), tanned (*n =* 32; 0.7%), and unknown (*n =* 1328; 30.1%). Circles represent archaeological sites, and their diameter reflects the sample size of brains at each site. (*b*) Number of preserved human brains through time by type (note the log-scale *y*-axis). Locally estimated scatterplot smoothing (LOESS) shows conditional means with 95% confidence intervals (CI). The saponified type is not shown, given insufficient data points (*n* = 7) for LOESS regression. (*c*) Ten types of human remains (*n* = 13 407) in the IsoArcH repository [7]. Mineralized collagenous tissues (i.e. bone [*n* = 8762] and dentine [*n* = 125]) represent the bulk of the database at 66.3% of total samples (*n* = 8887/13 407), followed by heavily mineralized (i.e. tooth of indeterminate subtype [*n* = 1720] and enamel [*n* = 1527]) and keratinous tissues (i.e. hair [*n* = 879], nail [*n* = 138] and beard [*n* = 4]) at 24.2% (*n* = 3247/13 407) and 7.6% (*n* = 1021/13 407), respectively. Non-mineralized collagenous (i.e. skin [*n* = 209]) and internal soft tissues (i.e. muscle [*n* = 34] and gut [*n* = 9]) together comprise a small fraction of the database at 1.9% (*n* = 252/13 407).

**Figure 2.**
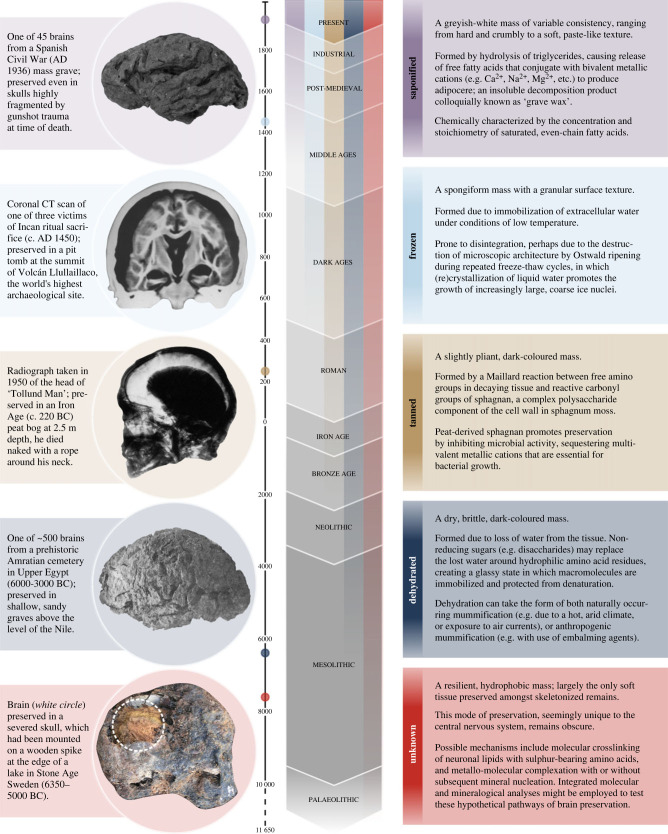
Morphological and biochemical features of five mechanisms of brain preservation. Images adapted from source material for saponified [8], frozen [9], tanned [10], dehydrated [6] and unknown [11] brains.

We identified a total of 4405 preserved human brains from 213 unique sources, which were reported from every world region except Antarctica (figure 1*a*; see electronic supplementary material, dataset S1). These brains are universally described as discoloured and shrunken (i.e. reduced in volume compared to the brains of living humans), albeit with reported variations in their degree of discoloration and shrinkage. For example, more than 500 brains from a Predynastic cemetery in Upper Egypt (~ 6150 years BP) were ‘as much as two-thirds of the original length of the brain, or they may have shrunk to less than half the length’ [6]. We identified five types of brain preservation (figure 2), of which dehydration, freezing, saponification, and tanning are well-characterized modes of soft tissue preservation in human remains [12]. However, there may be overlap between preservation mechanisms, given the complexity of decomposition as a system of interrelated biogeochemical processes [13], and the subjective nature of reporting in some cases. For example, brains exposed to a combination of low temperatures, low humidity, and circulating air currents (e.g. those found at high altitude) might be more accurately described as ‘freeze-dried’ than ‘frozen’ or ‘dehydrated’.

We have also designated a fifth mechanism, which does not appear to have been codified in the literature on brain preservation, as ‘unknown’. Almost one-third of reported brains (*n* = 1328; 30.1%) exhibit this fifth mode of preservation. A variety of depositional contexts are described, including tombs [14], burial mounds [15], shipwrecks [16], wooden [1] and lead [17] coffins, shallow [16] and mass [18] graves, fragmentary remains [19] and severed heads [20]. Although the presence of clay and iron are commonly noted in the sedimentary matrix (*n* = 20/69 sites; 30.0%), their abundance is not quantified. Brains of this unknown type almost exclusively (*n* = 1308/1328; 98.5%) constitute the only soft tissue preserved among otherwise skeletonized remains, which suggests there may be a preservation mechanism particular to the central nervous system.

The frequency of preserved human brains (figure 1*b*) through the Holocene Epoch (approximately the last 12 000 y BP) reveals a general trend of decreasing frequency with increasing archaeological age (i.e. *postmortem* interval). The numbers of frozen and tanned brains decrease more rapidly after approximately 500 and 1500 y BP, respectively, while the number of dehydrated brains declines more slowly over a longer period, around 4000 y BP. Saponified brains (*n* = 1308) are reported from just seven archaeological sites, comprising too few data points to meaningfully plot a distribution. By contrast, brains of the unknown preservation type are known to persist beyond 12 000 y BP (e.g. [21]; see electronic supplementary material, dataset S1).

To evaluate the abundance of preserved human brains relative to other bioarchaeological remains, we compared their occurrence with that of preserved human soft tissues reported in searchable databases. Internal soft tissues (representing muscle and gut only) comprise just 0.3% (*n* = 43/13 407) of human bioarchaeological samples in the IsoArcH repository [7] (figure 1*c*), which documents more than 400 archaeological sites across Europe and the Middle East from the Mesolithic (~ 9000 y BP) to the 20th century AD. By contrast, the number of preserved brains identified in the literature during the same time period is markedly higher (*n* = 3862), and far exceeds the number of heavily mineralized tissues represented in the repository (e.g. teeth [*n* = 1720], which have a high preservation potential given their inorganic mineral content and number of anatomical elements). Other archaeological databases (e.g. ARIADNE, ADS) contained no records of soft tissues. Similarly, only a minority of sources that describe the preservation of nervous tissues (*n* = 18/277; 6.5%) report the frequency with which these were recovered (i.e. the number of brains preserved as a measure of individuals excavated): although the average is approximately one in three (35.7%), there is considerable variation (2.5–100.0%).

**Figure 3.**
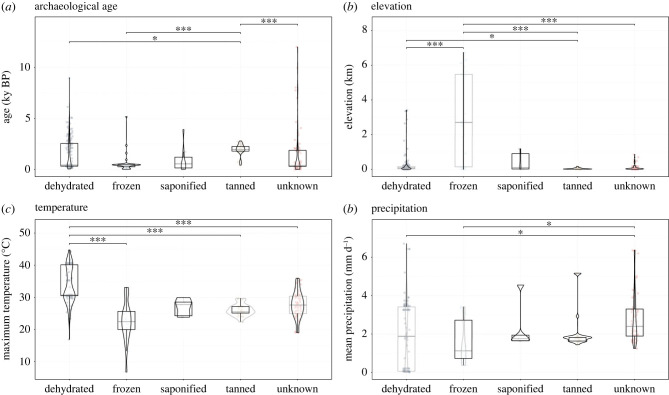
Relationships between depositional context and preservation type. Results of Kruskal-Wallis and *post hoc* Dunn tests with Holm adjustment for multiple comparisons at 95% CI, for preservation type with respect to (*a*) archaeological age, (*b*) elevation and the (*c*) mean monthly maximum temperature and (*d*) mean annual precipitation experienced from the time of death to time of excavation. Circles represent archaeological sites. Only statistically significant comparisons are shown, and indicated by the following significance codes: *** = *p*-value < 0.0001; ** = *p*-value ≤ 0.001; * = *p*-value ≤ 0.01. Results for latitude shown in figure 4*a*. Approximately one-quarter of terrestrial sites (*n* = 73/276; 26.4%) fell on grid cells with no climatic data and were excluded from the analysis (see electronic supplementary material).

**Figure 4.**
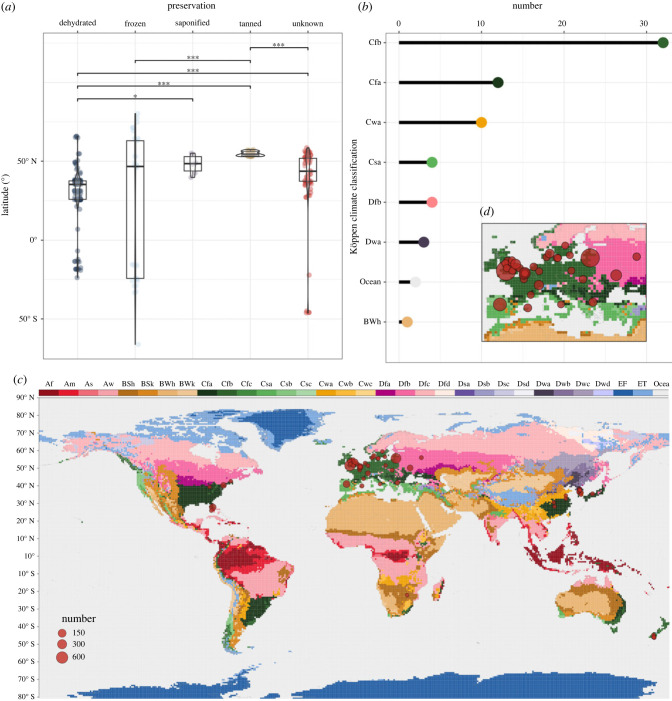
Relationships between geographical location and unknown preservation type. (*a*) Results of Kruskal–Wallis and *post hoc* Dunn test with Holm adjustment for multiple comparisons at 95% CI, for all preservation types with respect to latitude. Only statistically significant comparisons are shown, and indicated by the following significance codes: *** = *p*-value < 0.0001; ** = *p*-value ≤ 0.001; * = *p*-value ≤ 0.01. (*b*) Number of Köppen climate classifications based on data for the period AD 1986–2010 for archaeological sites yielding brains of the unknown preservation type. (*c*) World map of Köppen–Geiger climate data for the period AD 1986–2010 at 5 arc minute resolution [22,23], illustrating locations of archaeological sites yielding brains of the unknown preservation type. Circles represent archaeological sites, and their diameter reflects the sample size of brains at each site. (*d*) Distribution of brains of the unknown preservation type in Europe, where the majority (*n* = 40/69; 60.0%) have been excavated from locations principally experiencing an oceanic climate (Cfb; *n =* 29/40; 72.5%).

Using a Kruskal-Wallis and *post hoc* Dunn test with Holm adjustment, we identified statistically significant differences in terms of archaeological age ($\chi_{4}^{2}=24.86$, *p* = 5.42 × 10^−5^), elevation (i.e. height above sea level; $\chi_{4}^{2}=41.12$, *p* = 2.50 × 10^−8^), latitude ($\chi_{4}^{2}=86.32$, *p* < 2.20 × 10^−16^), maximum temperature ($\chi_{4}^{2}=84.50$, *p* < 2.20 × 10^−16^) and mean precipitation ($\chi_{4}^{2}=14.34$, *p* < .01) among all types of preservation (figures 3 and 4).

Dehydrated brains constitute the largest proportion of the dataset (*n* = 1667/4405; 37.8%), with several large assemblages of naturally mummified remains represented, including the aforementioned prehistoric site in Upper Egypt, containing more than 500 individuals ‘in every one of which the brain was preserved’ [6]. Over their taphonomic history, dehydrated brains experienced the highest maximum monthly temperature (mean [standard error of measurement, SEM] = 33.52 [0.48]°C), whereas frozen brains experienced the lowest (mean [SEM] = 22.68 [1.11]°C). Frozen brains are associated with the highest average elevation (mean [SEM] = 2920 [449] m) and, over their taphonomic history, the lowest daily precipitation (mean [SEM] = 1.57 [0.19] mm day^−1^): for example, victims of Incan ritual sacrifice at the cold, dry summit of Andean volcanoes [9]. Tanned brains are found at the lowest average elevation (mean [SEM] = 44 [7] m) and the highest average latitudes (mean [SEM] = 54.71 [0.26]°), comprising mainly Iron Age bog bodies from northern Europe (*n* = 23/32; 71.9%). Saponified brains cluster at mid-latitudes (mean [SEM] = 48.01 [2.34]°), but a range of depositional contexts are identified, including the waterlogged coffin of an infant interred at the confluence of the Rivers Odet, Steir and Jet in 13th C. France (∼ 688 y BP) [24], and rapid burial in a fire disaster following an earthquake during the Bronze Age (∼ 3900 y BP) in Western Anatolia, Turkey [25]. The vast majority (*n* = 1200/1308; 91.7%) of saponified brains, however, are reported from a single site: burials dating to the Middle Ages disinterred from Paris's largest cemetery prior to the French Revolution [26]. The contribution of this site to both the saponified type and the total dataset (*n* = 1200/4405; 27.2%) is substantial, and highlights the impact of reporting bias in the literature. Excepting this single context, saponified brains represent a small proportion of the archive (*n* = 108/3205; 3.4%).

Preservation types tend to correlate with a single present-day Köppen climate classification. Dehydrated brains are found principally in hot deserts (BWh; 58/136 sites, 42.6%), where high solar angles and nearly constant high-pressure systems create intense sunshine with little precipitation, producing some of the highest warm-month mean temperatures on Earth (generally 29–35°C) [22,27]. Similarly, approximately one-fifth of dehydrated brains (30/136 sites, 22.1%) are found in humid continental climates (Dwa), featuring hot summers and dry winters with warm-month mean temperatures above 22°C and winter precipitation below 10% that of the wettest summer month [22,27]. By contrast, frozen brains are largely confined to polar tundras (ET; 24/33 sites, 72.7%), typified by monthly mean temperatures below 10°C [27]. In these regions, low vapour pressure of atmospheric water results in little precipitation; however, evapotranspiration is equally low, allowing waterlogging of terrain.

Tanned brains are reported exclusively from oceanic climates (Cfb; 32/32 sites, 100.0%), which also host the most saponified brains (5/7 sites; 71.4%) and those of the unknown type (33/69 sites, 47.8%) (figure 4*b,c*). These climates are relatively mild year-round (generally 10–22°C), with no significant difference in precipitation between seasons [27]. A further one-third of brains of the unknown type are found in humid subtropical climates, which feature hot summers when precipitation peaks and storms reach the level of monsoons: 17.4% are found in regions that experience fully humid winters (Cfa; 12/69 sites), during which daily thundershowers remain common, while 14.5% experience dry winters (Cwa; 10/69 sites) [22,27. Accordingly brains of the unknown type are associated with the highest daily precipitation over their taphonomic history (mean SEM = 2.88 0.17] mm day^−1^).

## 3. Discussion

### (a) Geographical, climatic and temporal trends in brain preservation

The archaeological record preserves ancient human brains across time scales. Although these ancient brains are found worldwide, trends in their prevalence and persistence with palaeoclimate point to underlying environmental biases toward different types of preservation in space and time.

Saponified brains, however, are reported from too few sites to meaningfully probe these trends. Moreover, nervous tissues represent an unlikely substrate for preservation *via* saponification, which involves the formation of adipocere through the hydrolysis of triglycerides [28]. Adipocere forms readily from adipose tissue (the body's fatty, subcutaneous connective tissue), given it is composed almost entirely (> 98%) of triglycerides [29]. Although a lipid-rich organ, the brain contains less than 1% triglycerides [30]. This mechanism of preservation may be misidentified in brains reported as saponified.

It is perhaps not surprising to find that dehydrated brains are largely excavated from hot deserts, frozen brains from polar tundras, and tanned brains from temperate regions where bogs are widely distributed. That tanned brains and brains of the unknown type are largely confined to regions which, in AD 2000 (i.e. at the point of excavation, given a median date of source publication), experienced an oceanic climate may prove an artifact of excavation bias toward Europe. However, brains of the unknown type are also found in humid subtropical climates, similarly characterized by regular rainfall [31], in regions as disparate as North America, Asia, and Oceania. Unlike tanned brains, they are not confined to peat bogs and, most intriguing, are the only soft tissue remaining.

The features of the unknown preservation type appear to be reflective of a genuinely disparate mode of soft tissue preservation operating in diverse environments. Apart from similar climates, few unifying factors can be identified that characterize the depositional contexts in which preserved brains of the unknown type are found. That sites yielding brains of the unknown type are distinguished by the highest daily precipitation of all preservation modes runs contrary to the general taphonomic understanding that heavy rainfall increases the rate of decay [21]; although specifically subaerial decomposition (i.e. exposed soft tissues) may be slowed due to the suppression of insect activity [32], and the impact of precipitation alone may be difficult to disentangle from other environmental variables [33]. The anecdotal presence of clay and iron in sediments with brains of the unknown type might imply the importance of what is dissolved in the gravesoil by the action of rainfall [34]; however the effect of gravesoil chemistry and matric potential (i.e. water content) [35] on decay retardation, and particularly their broader associations with geography and palaeoclimate, remains little investigated.

Although there is a general trend of decreasing frequency of preserved brains with increasing archaeological age (figure 1*b*), differences in the rate of loss suggest differences in the persistence of preservation types. All mechanisms of brain preservation identified involve loss of water from the tissue, except for saponification (which involves its consumption). However, the sharp decline in the number of frozen brains after roughly 500 y BP, for example, compared with the more steady decrease in the number of dehydrated brains over approximately 4000 y BP, might suggest that removing extracellular water is more stable than immobilising it. Further, these trends suggest that ephemeral preservation of the brain on ≲4000 y time scales may occur where decomposition is arrested impermanently by transitory environmental conditions (e.g. temperature extremes facilitating dehydration or freezing) [36]: when the environmental conditions arresting these processes change (e.g. climate warming), decomposition proceeds and the tissues are lost. Meanwhile, brains of the unknown type persist for millennia in numbers that are orders of magnitude higher than what is observed for other preservation types.

### (b) Mechanisms of brain preservation on geological time scales

It is unclear what accounts for the unknown mechanism of preservation, in which the human brain persists on longer geological time scales (≳12 000 y; versus the maximum age of dehydrated: 8970 y, frozen: 5180 y, saponified: 3900 y and tanned: 2790 y), after other organs have perished. The skull might afford the brain protection from exogenous agents of degradation (e.g. animal scavenging, humidity/aridity, soil pH, etc.), akin to bone marrow shielded within the medullary cavity, the decomposition of which is inhibited where cortical integrity is maintained [37]. However, preserved brains have been discovered in skulls fragmented by both *perimortem* trauma [38] and taphonomic damage [39]. The persistence of the brain in this unknown type of preservation, irrespective of any protective action of the skull and in varied depositional settings, suggests some intrinsic quality associated with nervous tissue itself that permits long-term preservation.

**Figure 5.**
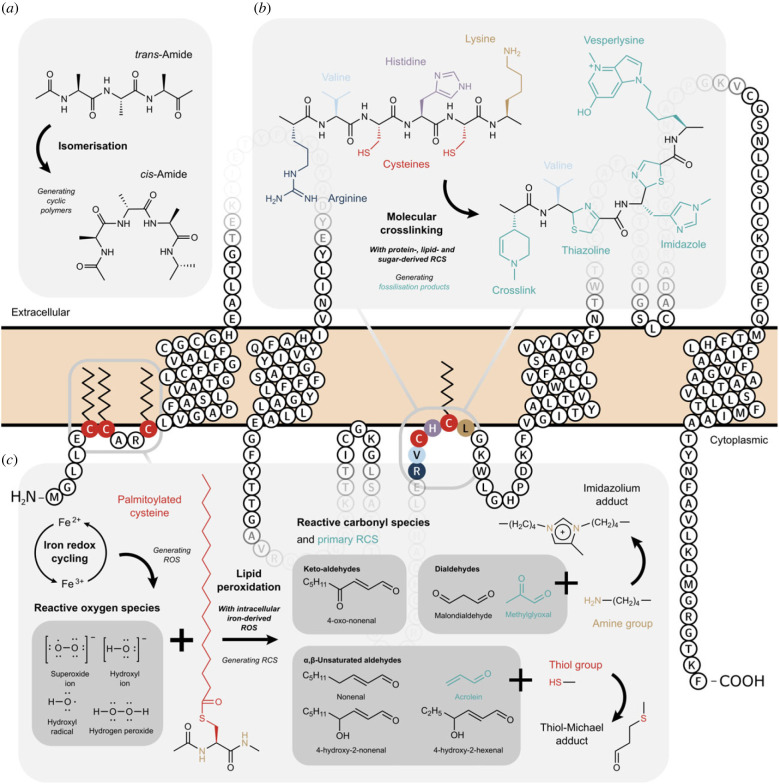
Chemical processes of molecular crosslinking and metal complexation potentially underpinning the unknown preservation mechanism. Proteoform of human myelin proteolipid protein (PLP1), the major transmembrane protein of the central nervous system [42], illustrating (top to bottom) the extracellular, transmembrane, and cytoplasmic spaces; created with Protter (v.1.0) [43]. (*a*) Covalent crosslinks formed during normal physiological processes within and between polypeptides are crucial for determining protein structure and function. However, unintentional crosslinking (e.g. *via* lipidation and glycation) converts intact peptide bonds (*trans*-amides) to *cis*-amides, as N-, O- and S-heterocyclic polymers are produced by isomerisation about the C–N bond. This cyclization results in a more stable molecule that might persist through time [41]. (*b*) Early crosslinking preferentially involves highly reactive amino acid residues. Arginine (R), cysteine (C), histidine (H) and lysine (L) form characteristic fossilization products *via* crosslinking with adjacent residues (particularly hydrophobic sites; e.g. valine [V]) or RCS [41]; a process that may be catalyzed by transition metals such as iron. (c) Intracellular iron redox cycling generates reactive oxygen species (ROS) that induce lipid peroxidation [44], which in turn yields RCS that become available for molecular crosslinking. Brain transmembrane lipids such as PLP1 feature multiple lipid-bonded, sulfurous amino acids (e.g. palmitoylated cysteine) [42], whose fragmented N- and S-containing functional groups react with primary RCS (e.g. methylglyoxal and acrolein) to form adducts [45]. These products then undergo condensation to form resistant, high molecular weight polymers that are stable on geological time scales [46].

Molecular crosslinking is a viable possibility for the unknown preservation mechanism. During fossilization of soft tissues, proteins, lipids and sugars converge in composition through lipidation and glycation to form chemically stable, polymerized macromolecules [40]. This process involves crosslinking between amino acid residues and reactive carbonyl species (RCS) formed in early diagenesis [41], the period after the death and burial of an organism (figure 5*a*). Thiol-bearing residues crosslink selectively in the presence of lipid-derived RCS and with adjacent hydrophobic residues [41]. With reactive, sulfur-containing side chains, thiol-bearing residues tend to be sequestered in the core of transmembrane proteins, which are tightly bonded to membrane lipids and feature a high proportion of hydrophobic residues [47] (figure 5*b*). The brain, which has the highest tissue-enriched expression of transmembrane proteins of any tissue [48], represents an ideal mixture of sulfurous, lipid-rich precursor reagents for this hypothesized pathway to fossilization.

The formation of inorganic phases, such as *via* metal complexation followed by mineral nucleation, that replicate or chemically stabilize nervous tissues, represents another possibility for the unknown preservation mechanism. Iron, the most abundant transition metal in living organisms [49], is especially abundant in the brain where it accumulates with ageing [50]. Its presence in association with preserved brains has been confirmed through histological analysis [18], and conjectured on the basis of macroscopic observations: for example, yellow to black surface colours of twenty-five brains from a medieval cemetery (∼ 522 y BP) in Hull [16]. The diversity of colours might suggest iron species in a variety of oxidation and coordination states; for instance, ferric (Fe[III]) oxides tend to be red-orange (hematite and ferrihydrite) or yellow (goethite), while ferrous (Fe[II]) sulfides (like pyrite) and ferrous-ferric oxides (like magnetite) are black [51].

In addition to forming inorganic phases, metals may enhance the potential for organic crosslinking, providing overlap between these possible preservation mechanisms. For instance, fossil melanic pigments from skin, eyes, ink and feathers dating to at least the Late Jurassic have been accredited to the crosslinked nature of the melanin molecule *in vivo* [46]. Alongside crosslinked peptide-lipid components, melanin comprises several polymers with negatively charged carboxyl, hydroxyl and amine groups that bind metallic cations to form stable complexes [52]. Heavier metals such as Fe(III) and Cu(II) bind most strongly and accumulate over time [53], and elevated concentrations have been reported in diagenetically-altered and fossil melanosomes [54].

Melanin is found in multiple brain regions as neuromelanin: a darkly pigmented, insoluble substance whose concentration increases with age. Neuromelanin is well-known for its affinity for iron, which it chelates and accumulates in various oxidation states [55]. Relatedly, diagenetic incorporation of sulfur in functionalized lipids (i.e. natural vulcanization) promotes the *in situ* polymerization of melanin [56]; this potentially rapid process (taking place within hundreds of years of deposition [57]) generates kerogens, recalcitrant macromolecules composed of long-chain hydrocarbons crosslinked by (poly-)sulfide bridges [58]. The sulfurous, lipid-rich brain may be especially susceptible to such a pathway to fossilization.

In contrast with diagenesis, one means of accumulating metallo-molecular complexes in the brain during life is disturbance of neuronal iron homeostasis [59]. While vital for healthy brain function, excess concentrations and certain oxidation states of iron may be toxic, which generate harmful free radicals known as reactive oxygen species (ROS) [59]. Under normal physiological conditions, neuronal iron is stored as the redox-inactive mineral ferrihydrite (ferric oxide) within the iron storage protein ferritin. However, when iron-protein binding is disrupted *postmortem*, ferrihydrite reduces to form a redox-active ferrous mineral phase, which may be subsequently oxidized to form magnetite (ferrous-ferric oxide) [44]. This redox cycling represents a ‘significant and sustained source’ of ROS that might continue to be generated *postmortem* [44]. ROS induce lipid peroxidation, which generates reactive aldehydes that can crosslink with thiol-bearing amino acid residues to form supramolecular adducts [60], analogous to those formed by RCS crosslinking (figure 5*c*). Moreover, the generation of nanocrystalline iron oxides dramatically accelerates the nucleation reaction of protein aggregates, which act as sites for further crosslinking [61]. The deep-time stabilization of proteins *via* iron-catalysed free radical reactions [40,62,63] has been argued previously to preserve endogenous amino acid residues from fossil soft tissues. Copper is another transition metal abundant in the brain and, with similarly variable valency to iron, might also participate in redox cycling of this type to complex with organics [64]. In fact, copper was first implicated in the preservation of ancient North American brain tissue over a century ago [15].

The interplay of crosslinking and complexation has been extensively investigated in the brain *in vivo* given its central role in brain ageing and neurodegenerative disorders [59]. For instance, clinical studies have probed neuronal protein retention as a function of temperature in the short (< 96 h) *postmortem* interval (e.g. [65]). Little is known, however, about diagenetic crosslinking of neuronal proteins on longer time scales. Mass spectrometric strategies for characterizing the nature and modification of protein-lipid [66], melanic [67] and metallo-molecular complexes [68] are well-established and provide a means to evaluate the crosslinked nature of preserved brains. In addition, non-destructive spectroscopic methods are well-suited for rapid, *in situ* characterization of molecular fingerprints [69] and their diagenetic alteration [41] in fossil organic matter, and constitute a valuable corroborative approach for precious, ancient samples. Finally, a range of largely X-ray-based methods might be employed to refine the identity [70], oxidation state [71], and tissue distribution [72] of metals and minerals intimately associated with preserved nervous tissues.

### (c) Palaeobiological applications of preserved brains

The record of preserved nervous systems is not confined to ancient human brains. In AD 1888, palaeontologist E. T. Newton (1840–1930) described a preserved brain within a new species of pterosaur from the Upper Lias of Southern England [73]. Numerous non-human brains have been identified in the archaeological and fossil records since. Among the youngest preserved brains are those of dozens of Antarctic fauna (e.g. seals [74] and penguins [75]) exposed in the polar desert, spanning roughly 100–3270 y BP. At the other end of the planet, Pleistocene mammals, including mammoth [76] and bison [77], entombed in permafrost in the Russian Arctic, have remained frozen over many millennia, up to around 45 000 y BP.

Among the oldest preserved nervous systems are those of early animals dating from the Cambrian Explosion, prior to 500 Ma [78]. By illuminating how nervous tissues first evolved, these ancient preserved tissues are proving useful in understanding where fossils are placed on the tree of life (e.g. [79]), and in reconciling fossil morphology with predictions from developmental biology [80]. Evolutionary insights like these are now possible precisely because of the morphological disparity of metazoan nervous systems, which are highly heterogeneous in their ultrastructure and function [81]. At the molecular level, however, their biochemical composition is relatively conserved [82,83]: neurochemical maps suggest homologies between the vertebrate and invertebrate nervous system ground plan [84]. This shared cellular foundation raises the possibility that the preservational mechanisms hypothesized above might operate in a range of taxa across the animal kingdom throughout the Phanerozoic.

Soft tissues are generally a richer source of biomolecular information than hard tissues *in vivo* [85], yet only a handful of preserved animal [86] and human [3,20,87–89] brains have been sampled for proteins, lipids and ancient DNA (aDNA). Among the human brains studied, the ‘Heslington’ brain (Yorkshire, UK) has yielded 783 proteins, the largest collection of ancient proteins (*palaeoproteome*) retrieved from any archaeological material investigated [89]. For comparison, around 100 proteins have been recovered from Iron Age dental calculus [90] and 15 from an Early Neolithic bone finger ring [91]. Preserved human brains, then, may constitute an exceptionally rich reservoir of ancient biomolecules, and their ubiquity in the geological record represents an untapped source of palaeobiological data. Below we outline specific areas of future research.

#### (i) Palaeopathology

Preserved nervous tissues could advance our understanding of the pathogenesis of neurological disease, particularly in ancient humans. Whereas methods for the study of brain ageing and neurodegenerative disorders in archaeological contexts remain limited, clinical neuroscience relies on the precise quantitation of a host of protein biomarkers for neurodegenerative processes, whose relative expressions are known to indicate disease progression [92]. Given the richness of human brain palaeoproteomes [3,89], it is possible that potential outliers in terms of protein expression and modification might be investigated as indicators of ancient neurodegenerative disorders. Similarly, the composition and distribution of diverse human brain lipids have been profiled for a variety of modern metabolic disorders [93]; particularly those involving disturbances in lipid homeostasis, such as diabetes and obesity. Preserved brain lipids may also provide insight into the nutritional status (i.e. the richness or poorness of one's diet) of ancient humans.

#### (ii) Archaeogenetics

aDNA recovered from preserved human brains might answer questions about our genetic history that the phylogeography of modern populations cannot address [94]. Over 30 years ago, the first attempts were made at retrieval and sequencing of aDNA from human brain tissue preserved ∼ 8000 y BP [95]. Since then, two studies of preserved human brains [14,96] have yielded promising results: Oh *et al.* [14] sampled aDNA from preserved brain and the associated bone, while Graw *et al.* [96] additionally sampled the associated teeth. The former reported markedly higher yields of less fragmented aDNA from the brain, while the latter recovered aDNA exclusively from the brain. In fact, genomic profiling studies [e.g. 97] of *postmortem* soft tissues suggest that the human brain yields the highest number of analytes and quality and quantity of DNA among organs sampled, even many decades *postmortem*.

Isolation of ancient chromatin (a complex of DNA and proteins forming chromosomes) from preserved nervous tissues might enable investigation of epigenetic patterns of aDNA methylation [98]. These heritable changes in gene expression are phylogenetically informative molecular targets that elucidate evolutionary trajectories [99] and ecological adaptations [100] at the population level, as well as permitting the study of phenotype (e.g. age at death [101] and morphological characters [102]) of ancient individuals.

## 4. Conclusion

The discovery of more than 4400 preserved human brains, with more than 1300 as old as 12 000 y and preserved in the absence of other soft tissues, suggests that nervous tissues persist in an abundance that has been overlooked in the archaeological literature. Our analyses reveal statistically significant differences in the geographical, climatic and temporal distribution of human brains preserved throughout the Holocene by ephemeral conditions that arrest their decay. Molecular crosslinking and metal complexation (with or without subsequent mineral precipitation) represent feasible, testable mechanisms by which labile nervous tissues might preserve brains on even longer, geological time scales.

The archive compiled here represents the first step toward a comprehensive, systematic investigation of ancient brains beyond 12 000 y BP, and is essential to maximizing the molecular and morphological information they yield as the most metabolically active organ in the body, and among the most commonly preserved soft tissues. Ancient brains may provide new and unique palaeobiological insights, helping us to better understand the history of major neurological disorders, ancient cognition and behaviour, and the evolution of nervous tissues and their functions.

## 5. Methods

For detailed methodology, see electronic supplementary material. We searched for literature reporting preservation of brain tissues in the archaeological record (electronic supplementary material, figure S1 and table S1) for common features of tissue appearance and depositional environment. We identified five types of brain preservation (electronic supplementary material, table S2) and calculated their frequency (i.e. number of brains preserved as a measure of individuals excavated; electronic supplementary material, table S3). We georeferenced each site using Google Earth Pro (v.7.3.6.9345), and assessed environmental conditions at each using estimates of palaeoclimate [103–105], before assigning Köppen climate classifications [22]. We computed summary statistics for archaeological age, elevation, latitude, maximum monthly temperature, and annual mean precipitation for each brain, grouping by preservation type (electronic supplementary material, table S4). These variables were tested for normality and homogeneity of variance (electronic supplementary material, table S5) using R (v.4.3.20) [106], and Kruskal-Wallis tests (electronic supplementary material, table S5) and *post hoc* Dunn tests (electronic supplementary material, figures S2–S6) were performed to probe significant differences between preservation types with respect to depositional context.

## Data Availability

The data that support the findings of this study are available within the article and its electronic supplementary material [107].
